# Ethical governance of AI-based humanoid carebots: the case for Ethics of Techno-care

**DOI:** 10.1007/s11019-025-10305-3

**Published:** 2025-10-29

**Authors:** Sivan Tamir

**Affiliations:** The Center for Health Law and Ethics , University of Haifa , 199 Abba Khoushy Ave, 3498838 Haifa, Israel

**Keywords:** Humanoid carebots, Autonomy, Deception, Ethics of care, AI ethics, Roboethics

## Abstract

The reality of ageing population, with loneliness among older people recognised as a public health concern, and a significant shortage of caregivers—call for techno-creative solutions. Artificial intelligence (AI)-based humanoid carebots (AIhCs) are one emerging solution, where care for humans is being outsourced to robots, in what we dub as “techno-care”. The humanisation of carebots, employing anthropomorphism to influence care-recipients’ trust in and compliance with AIhCs, is a key issue, uniquely involving deception to achieve care-related goals, which may violate care-recipients’ autonomy, among other things. This problem, and other autonomy-related issues, underscore the need for techno-care-specific ethical governance. We therefore set out to examine the appropriateness of existing ethical frameworks for governing AIhC design and implementation, focusing on autonomy and transparency, which are fundamental for AIhC ethicality. We intuitively start by considering the more novel frameworks of AI ethics and roboethics, then move to examine the well-established bioethics and ethics of care (EoC) frameworks. Our analysis concludes that no existing ethical framework adequately copes with the autonomy-related and other challenges of *techno-care* by AIhCs. We consequently suggest that a new derivative framework needs to be developed and applied to this setting—that of “*ethics of techno-care”* (EtC). We first distinguish EoC care setting and care-relationships from those of EtC, then offer a preliminary outline for this new normative guidance, comprised of the following key elements: human care-sensitive design, minimally-necessary deception, techno-care transparency, techno-care privacy design, techno-care autonomy, techno-care responsibility and accountability, and diversity and cultural sensitivity in AIhC design.

## Introduction

The reality of ageing population (Chan et al. 2024), and with loneliness among older people being recognised as a public health concern by the World Health Organization (WHO), growing to epidemic proportions with 1 in 4 adults across the world reporting feeling lonely (WHO n.d.; WHO 2021a; Maese 2023); coupled with a significant shortage of caregivers—call for techno-creative solutions. Care robots (carebots) or social robots, are a health/wellbeing technology designed to replace humans to some extent, as carers and companions for isolated people and vulnerable populations. The deployment of carebots is presently at the stage of concept studies in real environments (Müller 2020), such as hospitals and nursing homes. The next obvious step would be their deployment in the community.

Carebots come in a variety of guises, functions and agency levels. Some have monitoring and data collection functions (Yew 2021), some employ artificial intelligence (AI) to interact conversationally (Berridge et al. 2023), some are designed as Humanoid robots to convincingly imitate human likeness. Enthusiasts and sceptics alike, seem to assume that the not-so-distant robotic era will see humanoid robots that are fully autonomous, appearing, communicating, and functioning like real humans (Kim and Kim 2022).

The practice of humanising robots employs Anthropomorphism, essentially attaching human features to non-human entities (Liu 2021). Reportedly, recent humanisation efforts have already succeeded in binding engineered skin tissue to humanoid robots’ faces, using cultured skin equivalent, allowing it to achieve biological functions of human skin with the capacity for self-repair and for expressing smiles (Kawai et al. 2024).

Some scholars hold that humanoid robots are intentionally developed to *replace* humans (Kim and Kim 2022), but humanisation efforts, at least initially, are more likely aimed at promoting human acceptance and trust in machines to *assist* humans. This is particularly significant in healthcare and welfare settings, where AIhC’s anthropomorphisation is instrumental in creating an illusion of social bonding between robots and humans, allowing better communication and interaction with care-recipients, facilitating cooperation with caregivers and improving care compliance (Langman et al. 2021).

AI-based humanoid carebots (AIhCs) are currently implemented as hospital receptionists to disseminate information; in radiology departments, to reduce risk for human operators (Carleo-Evangelist 2022); and as assistive nursing support, performing various tasks and monitoring patients. For example, Moxi, an AIhC able to form an emotional connection with patients through facial expressions—deployed in a COVID-19 ward for delivering and retrieving medical instruments, specimens etc. (Ibuki et al. 2023); and Grace—an AIhC with Asian features, specialising in senior care, uses a thermal camera to take patients’ temperature and measure their responsiveness, with capacity for social interaction and speaking three languages (Reuters 2021). AIhCs are also used as social companions, assistants for elderly, and for teaching children and people with disabilities, e.g., Milo—a humanoid teaching robot able to make a range of facial expressions to help children with autism identify human emotions and develop social skills (Goodman 2018; Robots4Autism n.d.).

In this paper, we focus on AIhCs, broadly defined to include companion robots. We are specifically interested in AIhCs with self-learning capabilities and (semi-)autonomous decision-making, that replace human caregivers/companions in performing care-related tasks. The human-like features of AIhCs, causing deception, presents an interesting case for their deployment among populations with vulnerable attributes (e.g., illness, solitude), or situated in vulnerable settings (e.g., hospitalisation or living in a health care facility), such as older persons, people with diminished cognitive capacities due to neurodegenerative diseases, and children (Veruggio et al. 2011; Fiske et al. 2019).

The social pervasiveness of AIhCs in health- and social care, is likely to grow in view of several key factors: the continuous need of improving healthcare by adopting technological advances fit for purpose; the constant shortage in medical personnel; the said “loneliness pandemic” and the prevalent social isolation in modern society—both carrying adverse health impacts (Sweet 2021; NASEM 2020). This phenomenon, calls attention to human-robot interactions (HRI). These gain particular attention when it comes to said vulnerable populations.

As with any new technological development challenging existing ethical thought, the emergence of AIhC tests (and defies) current ethical frameworks. Looking into humanised robots’ inherent and instrumental deception, we consider the challenge it creates for care-recipients’ autonomy, under the more novel frameworks of AI ethics and roboethics—branches of applied ethics addressing AI and robotic technologies, yet under development, as well as under the well-established ethical frameworks of bioethics and ethics of care.

Despite some suggestions for ethical frameworks for the design of carebots (e.g., Sorell and Draper 2014),^1^ we find that, to date, no existing, established, ethical framework adequately copes with the specific (autonomy and transparency-related) challenges of AIhCs. We therefore suggest that as care for humans will gradually be outsourced to robots and smart technological devices, a new derivative framework needs to be developed and applied to this setting—that of *“ethics of techno-care”*. But, at the outset of our quest for an appropriate ethical framework, we shall first introduce what is good, bad or disconcerting about AIhCs.

## AIHCS—Benefits, limitations and concerns

The literature cites various benefits for using AIhCs for care of the said target populations. A non-exhaustive list of techno-pragmatic and socio-personal benefits, can be roughly classified into the following categories:

1. Accuracy and reliability. AI’s outputs, translated into AIhCs’ functioning, are the product of analytics of vast amounts of data and are therefore more accurate than human-care-providers’ judgements. This, in turn, indicates greater reliability of AIhCs, and increases the confidence of deployers and [cognitively aware] care-recipients in the robot (Kim and Kim 2022);
2. Personalisation—enabled by AIhCs’ dynamic development and gradual adaption to care-recipients’ particular personality and needs—creating optimal engagement with the AIhC for the care-recipient;
3. Dignifying engagement and robot forbearance. Importantly, AIhCs can stimulate cognitive engagement with care-recipients, using conversational functions, while maintaining their human dignity, e.g., by tolerating repetition that is typical for some cognitive impairments and avoiding (human) caregivers’ irritated responses (Berridge et al. 2023);
4. Translatability. The designed human-likeness (elaborated on below), as well as human-like reactions of AIhCs could promote the translatability of robotic interventions to improve HRI and care-recipients’ trust, and ultimately, to better human welfare;
5. Personal autonomy. Deploying AIhCs in the community, in elderly people’s homes, can help them maintain their independence for a longer period of time and remain rooted in their communities and in their natural environment (Coin and Dubljević 2021), thus respecting their preferences for autonomous living at home;
6. Replacing human labour. Purportedly, AIhCs could mitigate the acute shortage of human resources in healthcare and social care. Although, this promise appears limited in reality. Take Japan—a seemingly ideal setting for carebot deployment, with its strong technology-enthusiast culture, growing elderly population, and a continuous drop in caregivers. Wright, who conducted a study at a nursing care home in Japan that was trialling carebots, somewhat curbs carebot-enthusiasm, at least for the near-future. His conclusions were that ultimately, the machines failed to save labour and that a growing body of empirical evidence finds that robots tend to *create* more work for caregivers. Interestingly, referring to the nature of the new caregiving work modality, Wright observed that existing social and interactive tasks were replaced by new tasks involving more interaction with the robots than with the care-recipients (Wright 2023);
7. Improved physician-patient relationship. Techno-positivists envision that introducing AIhCs into healthcare and social care, could assist healthcare professionals with patient communication, and improve patient engagement, consequently reducing caregivers’ burnout (Coin and Dubljević 2021) and healthcare costs, allowing greater accessibility to such services; and
8. Augmented intelligence. This is another professional promise, typically cited by AI-enthusiasts, suggesting that humans working alongside AI-based technologies benefit from having their intelligence increased by such interaction (Tamir 2023). This, however, is not axiomatic and worthy of empirical research.

Sceptical and critical attitudes towards AIhC implementation, are equally rich, and can be organised into the following categories:

1. An epistemic limitation. Our understanding of AIhCs suffers from knowledge gaps, due to having too little evidence, gained through very limited experience with them (Berridge et al. 2023). More research is needed to inform stakeholders (including policymakers) about the contribution and perils involved in deploying AIhC to care for vulnerable populations, including potential effects on identity, agency, and self-consciousness in patients (Fiske et al. 2019).
2. Depersonalisation of care and reduced human interaction. AIhC-sceptics, generally display reluctance towards the widespread deployment of AIhC to form human-like connections with target populations. Since robots are non-sentient machines, AIhCs cannot perfectly imitate human-human interactions, inevitably creating an authenticity-gap. They express fear of potentially reduced opportunities for human interaction (Sparrow and Sparrow 2006) and for practicing and cultivating empathy (Vallor 2011); and dread the *depersonalisation* of care, in the sense of forgoing physical and relational human touch and meaningful connection. Although, admittedly, it has been argued that in some care contexts, care-recipients may prefer interaction with a neutral robot, which could be more constructive for maintaining their human dignity, than engaging with (un)familiar human caregivers (van Wynsberghe 2013). This, however, may not necessarily be the case for populations suffering from loneliness/isolation, which could be better mitigated by authentic human contact. AIhCs incapacity of authentic human care, including (non-)mutuality, leads to broader concerns for a dystopian future where care is de-humanised (Müller 2020; Yew 2021; Kim and Kim 2022; Berridge et al. 2023). Conversely, people tend to grow accustomed to the presence of technology in most areas of life. So, we suggest that a mitigating argument here is what we term as “*inevitable habituality*“, or “*exposure-based optimism”*. Kim and Kim (2022) submit that with more frequent, common exposure to robots in healthcare settings, people’s confidence in robot-based services, will increase.
3. Dignitary and personal harms. This category encompasses concerns about objectification of the elderly (or other care-recipients), emotional harm (when realising that one is interacting with a machine rather than a human caregiver), social harm (if care-recipients grow to prefer their robot companion over their relationships with fellow humans) (Sharkey and Sharkey 2021), and confusion and overreliance of care-recipients.
4. Deception,^2^ which is also a dignitary harm, is a category in itself and a central theme in this paper. It is a widely cited ethical concern in the context before us– one of the tacit purposes of designing and developing humanoid robots. AIhC-deception may cause care-recipients to mistake robots with human-like appearance and behaviour, for genuine people, entrusting them with their care, and falsely believing that AIhCs are sentient creatures who can reciprocate attention, love, understanding and authentic companionship, thus harming their dignity. This is particularly disconcerting for older, less technologically literate individuals, and for cognitively impaired and otherwise vulnerable people.^3^ Deception also potentially violates care-recipients’ autonomy—our topic of focus—in several ways: One, in the sense of (not) having control over one’s life, through receiving true knowledge to inform choices (e.g., consenting to being cared for by a robot). The other, corresponds with Ruddik’s (1999) definition of autonomy, as “the capacity to lead a chosen or embraced life”, or “the ability to *continue*,* so far as circumstances allow*, the life one embraces [emphasis added by author]”. Accordingly, deception may also undermine the vulnerable care-recipient’s right to self-determination as an autonomous person with specific preferences.
  We are cognisant that while deception carries a negative connotation, and stands in stark contrast to the principle of transparency in the sense of honesty—unmasking the humanoid robot as a mere machine would be counter-indicative, conflicting with the semblance of humanity AIhC developers aim to achieve to gain care-recipients’ trust and compliance, *for the latter’s own good*.^4^
  Yew (2021) maintains that deception should be considered morally wrong, by default, due to deontological grounds (e.g., respect for humanity) and because it offends the virtues of honesty and trustworthiness. In the same vein, Sparrow and Sparrow (2006) argue that deception goes against our duty “to see the world as it is” and it is bad because “our preferences are unlikely to be met, our interests advanced, or our wellbeing served, by illusions.” They further contend that deception uses manipulative objectification, violating the fundamental Kantian duty to respect people as ‘‘ends in themselves’’; and that even if it is employed for the subjective benefit of the care-recipient—the *intention to deceive* (even if well-meant) is unethical, particularly if its result is harmful to the former.
  Consequentialist views of robotic deception maintain that deception is wrong when it creates harmful impacts on individuals and society. In the context before us, inappropriate use of robots to replace human care and misplaced trust in carebots’ decision-making capabilities, will result in an overestimation of the robot’s functionality or in an illusion of sentience or cognition (Sharkey and Sharkey 2021). Deception is also wrong when it is intentionally designed for unethical reasons, carrying negative consequences that misalign with care-recipients’ best interests, and deployer-user trust is breached (Grodzinsky et al. 2015). Appropriately, where developers’ intentions are good, e.g., if benign deception (that does not constitute a breach of trust) is employed in the robot’s design, with good consequences (better outcomes) for the deceived—some deliberate manipulations causing deception, are ethical (Grodzinsky et al. 2015; Sharkey and Sharkey 2021). Ergo, the ethicality of AIhC-deception ought not consider harms alone, but also navigate ethical trade-offs, such as AIhC-care benefits (cited above).

5. Technical concerns and professional scepticism. A prominent doubt and technological hurdle frequently raised by sceptics, relates to “teaching” AIhCs human-specific gestures and communication skills, given the limitations of current AIhC algorithms in replicating inter-relational qualities (e.g., body language, human voice, emotions), or intuition (Coin and Dubljević 2021; Kim and Kim 2022; Berridge et al. 2023). We shall dub this, the “*you-cannot-program-humanness-argument”*. Also, a systematic literature review of nursing staff’s attitudes, needs, and preferences related to the use of carebots in assisted living facilities (Trainum et al. 2024), found that while carebots’ assistance with physically demanding tasks to reduce workload was positively viewed—nursing staff had mixed feelings on carebots’ assistance with social tasks. Although the reported studies used relatively small sample sizes (and therefore suffer from limited generalisability, among other things), they largely indicate that nursing staff mostly believed that delegating social support to a robot, would have serious implications. They were concerned about the ethics of carebots (e.g., maintaining the older adults’ autonomy and dignity); their safety (in terms of staff’s supervision and having ultimate control over the carebots); operability (carebots may require high-maintenance and consequently increase workload; and a related concern about the absence of education and training on care technologies in nursing curricula); reliability; and accessibility (in terms of distributive justice).
6. Pragmatic, forward-looking concerns. These primarily address human-redundancy in healthcare (e.g., nursing staff’s apprehensions about being replaced by carebots, and losing their jobs to them) (Trainum et al. 2024), particularly in typically feminised jobs. This will probably have broader, cross-societal socio-economic implications, affecting other professionals in the health and social care sectors, blue-collar workers, in particular. An interesting, human-redundancy-related argument heralded by Vallor (2011) and addressed by others (Rhoads and Marsh 2023; Yew 2021; Coin and Dubljević 2021), claims that the implementation of AIhCs may deprive value from human caregivers, in terms of quasi-altruistic goods, e.g., job satisfaction, and the promotion of their subjective well-being; and goods central to care practices, such as reciprocity, empathy and emotion.
7. Prohibitive cost. Another pragmatic concern is the considerable cost of creating a single AIhC and its expensive maintenance. These may render training and employing human personnel, more cost-effective and realistic. Such cost, however, could be reduced over time.
8. Informed consent to AIhCs’ care. This ethico-legal concern about obtaining informed consent of people with diminished cognitive functions for AIhC interaction, will be addressed under the section on bioethics.

We now turn to analyse the appropriate ethical landscape for AIhCs, examining what ethical framework can adequately address the ethical, autonomy-related and other implications of deploying AIhCs.

## What ethical framework should govern AIhC implementation?

Several ethical frameworks may apply to settings involving AI-based healthcare technologies and human patients: the well-established bioethics and the normative ethical theory of ethics of care (EoC), AI ethics, and more specifically—Roboethics. Concededly, the two latter ethical frameworks, each in their own domain, do not constitute a single uniform framework (Qiang et al. 2024), but they do share common core principles, reiterated in any ethical framework suggested, respectively, for AI and robotics (European Parliament 2019). We would argue that in their present form, none of these frameworks is suitable for the design, development and implementation of AIhCs; and a new designated framework, drawing on specific principles from existing ones, ought to be applied here.

We shall start with the intuitive candidates, i.e., the two latter frameworks (AI ethics and Roboethics), and then move to more elaborately consider bioethics and EoC, focusing on autonomy, as well as on transparency (as instrumental for maintaining the care-recipient’s autonomy and human dignity), which are fundamental for AIhC ethicality.

### AI ethics

The field of AI ethics is ongoingly developing, with a variety of frameworks for adoption. A significant overlap has been identified between bioethics and AI ethics principles (Floridi and Cowls 2019), comprised of beneficence, non-maleficence, justice and autonomy, with the additional AI-specific principle of explicability (i.e., accountability for AI outputs and intelligibility of the inner workings of AI systems). However, as opposed to bioethics, AI ethics principles are typically general (interpreted contextually according to their specific application), representing abstract values, and featuring a *humanity-centred* (rather than patient-centred) approach. They are consequently more systemic, seeking to ensure safe and responsible AI design to protect the interests of humankind from AI’s ‘antagonistic’ capabilities. For example, the European commission High-Level Expert Group on AI (2019) *Ethics Guidelines for Trustworthy AI*, include transparency among its principles, alongside other relevant principles, such as: human agency and oversight; technical robustness and safety; privacy and data governance; diversity, societal well-being and accountability.^5^

An additional layer of healthcare-specific AI ethics frameworks, is also emerging. The WHO (2021b) guidance on *Ethics and Governance of Artificial Intelligence for Health*, identifies universal ethical principles, including autonomy, importantly asserting that “AI technologies should not be used for experimentation or manipulation of humans in a health-care system without valid informed consent”. This is highly relevant to the deployment of inherently deceptive AIhCs for interacting with vulnerable populations.

A more recent example is the American Medical Association’s (AMA 2023) *Principles for Augmented Intelligence Development*,* Deployment and Use*. A particular reference to transparency is made in these principles, establishing guidelines for *when* and *what* to disclose in using augmented intelligence-enabled systems and technologies. Remarkably, while these emphasise the need for the use of AI-based technologies in healthcare to be transparent *both* to physicians *and patients* (pointing to the essentiality of transparency for establishing patient-physician trust)—when considering “what” to disclose—the guidelines refer solely to the physician, paternalistically overlooking the patient. As with the WHO (2021b) principles, the transparency required here is more developers- and deployers-facing, than patient-facing. This principle is therefore not a good fit for the transparency dimension required for realising care-recipients’ autonomy, where implementing AIhCs in healthcare and welfare settings. [Oddly, the concept of autonomy is entirely absent from the wording of the AMA (2023) principles.]

As Tamir (2023) argues, whereas AI ethics principles, representing a human-centric approach, ought to be applied to the general framework and technological architecture of the AI tool—they fail to specifically apply to patient/care-recipient-centric personal and societal aspects, such as those arising from the implementation of AIhC in healthcare and welfare circumstances. Consequently, AI ethics principles alone are insufficiently appropriate for the design, application and use of AIhCs, interacting with patients or vulnerable populations. Notwithstanding this, AI ethics principles are uniquely apt to guide user-related technology design, and the responsibility and accountability for harms emanating from faulty/negligent AIhC implementation.

We now move to consider whether the framework of roboethics, is appropriate for AIhC.

### Roboethics

The advent of increasingly sensitive HRIs, and the tensions and new vulnerabilities these will create for human society, have led to the development of roboethics—a sub-field of applied ethics, more specifically—machine ethics. It is essentially a code of conduct for robot developers and implementers to follow, to ensure that autonomous robots will exhibit ethically acceptable behaviour within HRIs, or where robots’ actions may carry negative consequences for humans or the environment (Veruggio et al. 2011). Roboethics is mostly focused on robots as social actors.

The mentioning of roboethics principles typically starts with a reference to Isaac Asimov’s (1950) famous “three Laws of Robotics”.^6^ This is in fact the prototype for later roboethics principles, typically centered on “[human and humanity’s] safety, [robot] obedience, and self-preservation [of the robot]”, with the aim of setting boundaries between humans and robots (Langman et al. 2021).

On its face, while current roboethics principles seem to echo some version or another of Asimov’s laws, the application of these laws (devised in 1942) to more sophisticated contemporary technologies, implementing AI-based robots in different areas of life and creating novel situations of HRI—seems to lack the specificity and sensitivity required for such settings (Langman et al. 2021). As Murphy and Woods (2009) astutely describe it:

“Asimov’s laws assume functional morality…and ignore the legal and professional responsibility of those who design and deploy them… Asimov’s laws ignore the complexity and dynamics of relationships and responsibilities between robots and people…”.

And so, into this lacuna enter scholars (Pasquale 2021), industry, professional associations, international organisations and governments, to formulate policies, ethical frameworks and legislation, for optimal HRI. Notably, although a quasi-independent field—roboethics, particularly for AI-based robots, is oftentimes intertwined with AI ethics frameworks.^7^

#### AIhC autonomy—through the lens of roboethics

Focusing on the relations between AIhC-deception and care-recipient autonomy, we realise that the term “autonomy” under the framework of roboethics, is differently construed from “autonomy” under bioethics and other patient-centric frameworks (discussed below).

‘Autonomy’ in the sense of roboethics, typically applies to **artificial autonomy**, that of the robotic system. Namely, the extent to which an artificial agent, can operate independently, without direct human intervention/instruction. For AIhCs, we are particularly concerned with artificial autonomy that goes *beyond* the execution of actions predefined by the designer (Beghili et al. 2024), emphasising the robot’s operational and decisional independence from human beings [44]. In fact, roboethics aims to balance artificial and human autonomy (in the sense of control) (Langman et al. 2021). That is, how far can a robot’s unsupervised operation and decision-making go, without diminishing human autonomy, within HRIs.

Also, Murphy and Woods’ (2009) alternative Laws of Responsible Robotics speak of a “requirement for responsiveness”, as a new form of autonomy in the sense of appropriately engaging with others. They also recognise the formation of “new control relationships” between robot and human, and the need for unhindered transfer of control from the former to the latter, in specific situations. These roboethics principles, are more control-oriented, and fail to capture human autonomy-related aspects, in the context of healthcare and welfare.

A clear distinction between autonomy for robots and for humans, can be found in the European Group on Ethics in Science and New Technologies (EGE) (2018) *Statement on artificial intelligence*,* robotics and ‘autonomous’ systems*. The statement puts forward that as autonomy habitually involves self-awareness and self-consciousness, it can therefore only be attributed to human beings. Consequently, applying the term ‘autonomy’ to mere artefacts (even if highly advanced or intelligent systems), is a misnomer.

So, roboethics’ distinct applicability to artificial autonomy (rather than human autonomy), and the narrow sense of such autonomy, present a tangible limitation for applying roboethics principles to ethically guide or evaluate AIhC-deception, particularly as it relates to care-recipients’ autonomy.

Conversely, roboethics is a robot-specific ethical framework. See, e.g., the EGE (2018) statement calling for human dignity, which is particularly relevant to AIhC-deception:

“…there have to be (legal) limits to the ways in which people can be led to believe that they are dealing with human beings while in fact they are dealing with algorithms and smart machines. A relational conception of human dignity… requires that we are aware of whether and when we are interacting with a machine or another human being…”.

Nevertheless, despite being robot-specific, we would argue that on top of our autonomy-related arguments above, roboethics frameworks are simply not entirely adequate or sufficient, for AIhCs. For example, the European Parliament (2019) policy on AI and robotics, draws heavily from bioethics principles, ethical principles protected in the Treaty on European Union and in the Charter of Fundamental Rights, values of Union law and AI standards, and more (European Parliament 2019, § 147 & § 149]. This, in itself, is a strong indication of the insufficiency of roboethics frameworks for independently guiding ethical AIhCs design, development, and use.

Another case in point, is that roboethics principles are more oriented towards general AI applications (e.g., cybersecurity), and less appropriate for narrow AI, particularly in healthcare and welfare contexts. They also seem unsuitable to address human-care-related ethical aspects. Moreover, roboethics principles are mostly about *drawing boundaries* between robots and humans within HRI (Langman et al. 2021), rather than about AIhC-human *engagement* and [artificial] *relationship*, which is fundamental for healthcare and welfare.

After considering the suitability of AI ethics and roboethics frameworks for AIhCs, we now move to look at bioethics, with its EoC branch.

### Bioethics

Introducing AI and robotics into human care, presents new interdisciplinary challenges for bioethics. Intuitively, the patient or care-recipient-centric framework of bioethics, seems fit for purpose for AIhC settings. We shall first examine this intuition with respect to our principle in focus—autonomy.

#### Autonomy and informed consent

Even though AIhCs are essentially more wellness-oriented than therapeutic, we are nonetheless dealing here with a quasi-treatment (care and companionship). Obtaining care-recipients’ informed consent for the implementation of and interaction with such care-technology, is therefore a *sine qua non* condition for ensuring their autonomy. Also, practically, if placed on a technological continuum—we will find that even remote, less intrusive health-monitoring wearable devices, require consent to their terms of use.

Proper informed consent for AIhC use, requires transparency, supplemented by educating care-recipients on AIhC purpose, capabilities and functions, and the risks they pose (Wright 2023). Another informed consent-related point, is that beyond promoting care-recipients’ autonomy, in the sense of having control over their lives through informed choices—such as: Do I want to live independently? What kind of assistance would I be willing to get, and what kind of presence in my home—human or robot—could I tolerate?—informed consent also has important participatory purposes, namely, allowing care-recipients an active role in their treatment decision-making (Avci 2023).

So, within the constructs of autonomy, consent for being cared for by an AIhC should be sought from those capable of giving it. The care-recipient’s agency is naturally a factor. Part of AIhCs’ target population will be (temporarily, or permanently) lacking capacity—as in the case of cognitively impaired individuals. With full-fledged autonomy being unachievable for such individuals—proxy/surrogate decision-making is required. Such decision-making ought to be guided by and restricted to the care-recipient’s substituted judgement. Namely, their preferences, according to previously stated volitions, their values, and their best interests, considering their specific physical and emotional needs (Avci 2023). Also, informed consent-related transparency, requires that appropriate disclosure about AIhC-care, and explanation about AIhC-interaction and operative features of the system be provided to care-recipients, or to their surrogate decision-makers.

The question of agency for people with dementia, intertwined with respect for their autonomy, is a case in point. For these care-recipients, loss of personal decision-making capacity is progressive, advancing over time rather than instantly. Their capacity, therefore, needs to be temporally (re-)evaluated. To the extent that such faculties are preserved (reappearing when being consented), their consent should be sought, and their autonomy—in terms of respecting their values and volitions vis-à-vis being cared for by AIhCs—respected.

Another issue relating to informed consent and AIhC’s target population, is that even cognitively intact care-recipients (e.g., socially isolated individuals) may struggle with the epistemic prerequisites of informed consent, given the novelty and the highly technological nature of the application before us, and due to potentially insufficient technological literacy.

In this context, some contend that the measure of capacity required for consent, should be correlated with the level of risk involved. Namely, whereas highly risky interventions require greater decision-making capacity—a lower level of capacity could be satisfactory for less risky medical interventions (i.e., the sliding scale strategy) (Avci 2023). Arguably, companionship and monitoring by AIhC falls into the latter category. Alternatively, it creates a category of its own, given the non-pure therapeutic nature of AIhC-care, and its benign risks for autonomy and privacy. However, the underlying element of deception, inherent to AIhC, might require higher level of competence to see through the carebot’s masquerade, in order to provide free autonomous consent to care. Trickily, such requirement could be self-defeating, conflicting with the rationale for using AIhC, and undermining care-recipients' trust in the carebot. This could imply a shortcoming of patient-centered bioethics, which is inadequately equipped for considering the technological architecture of a given technology.

Lastly, as mentioned before, when vulnerable populations interact with AIhCs, their human dignity is violated to some extent by being deceived about who/what they are actually interacting with. So, their already susceptible autonomy is intertwined with an infringed human dignity. This, of course, is limited to patients with impaired cognitive capacity.

Now, while bioethics relevantly provides us with the key principle of personal autonomy, we may fare better by employing a more specific theory of normative ethics, for the particular context of caring for humans—that of care ethics. We shall now explore the potential suitability of this ethical framework.

### Ethics of care

Famously articulated by Carol Gilligan and Nel Nodding, the EoC moral theory emphasises caring, interpersonal relationships and relational responsibilities, and looks at the well-being of caregivers and care-recipients, in a contextual way, with sensitivity and empathy as necessary virtues (Sander-Staudt n.d.). Gilligan frames EoC as “the premise that as humans we are inherently relational, responsive beings and the human condition is one of connectedness or interdependence” (Interview 2011). Tronto (1993) further formulated the EoC, identifying the following ethical elements: attentiveness (caring about, noticing the need to care), responsibility (for care), competence (for caregiving) and responsiveness (of the care-recipient, to the care received).

Yew (2021) advocates that EoC is the most suitable ethical framework to guide carebots design. van Wynsberghe’s (2013) too, offers an approach of *value-sensitive design* for carebots, relying on the fundamentals of EoC. We would argue that although intuitively applicable to our sensitive setting involving (AIhC) relationships with vulnerable populations—EoC theory would be ill-suited here, for several reasons.

Firstly, a theoretical reason presented by van Wynsberghe (2013), according to which, EoC was developed “as a perspective or orientation from which one begins to theorize rather than a pre-packaged ethical theory.” Namely, EoC was never presumed to be ripe for a particular application. Instead, it was meant to construct a non-specific care orientation.

Also, the EoC framework, constructed long before the emergence of AI-based healthcare and welfare technologies, was not designed to contemplate care by robots and so, naturally, does not consider the provision of “technological care” via AIhCs. It draws on familiarity with and socio-moral expectations for human-human interaction in the context of care. As, unlike AIhCs, human caregivers are not a product or object of design (the equivalent for humans could be vocational education and training), EoC does not appropriately apply to the issues of responsibility for the ethicality and safety of AIhC design, nor to technology-architecture requirements for transparency, or to explainability aspects.

Secondly, a conceptual reason with practical implications: The conceptualisation of *caring* was never developed to fit caring by *non-humans*. EoC is grounded in human nature and human connectedness and appropriately features an anthropology-based dependence (Maio 2018). It concerns people caring *for* and *about* people, within the constructs of human-to-human interpersonal relationships, in an attentive, reciprocal, responsive, human-sympathetic manner, while employing emotional knowledge. Another element is the *motivation* to care for dependent and vulnerable individuals, which is “inspired by both memories of being cared for and the idealizations of self” (Sander-Staudt n.d.), and in this sense, is simply non-existent for robots. AIhC “motivation” to care, is nothing more than a technical stimulus programmed into the robot.

The question we should ask ourselves then, is whether AIhCs can actually *care* for humans, in the sense put forward by EoC theorists. That is, could AIhCs notice the need to care for specific care-recipients, and could they authentically CARE about them, in an attentive manner? Or, perhaps, AIhC would feature *techno-*attentiveness, merely fulfilling the technical elements required in care. Namely, imperfectly imitating human care, so that sympathetic reactions may come across as “synthetic” outputs, rather than real human-like attention, and with limited capacity for genuine reciprocity, which is “the seed of human sociality” (Vallor 2011). Will AIhCs be responsible for the care provided by them (or will responsibility lie with their developers/deployers, or the robot itself as a potential legal entity)? Also, lacking the neurobiology for attachment and bonding, and the biology-dependent capacity for complex social relationships (Sharkey 2017), do AIhCs possess the versatile range of competencies required for caregiving? On this point, there seems to be an instrumental limitation: while an AIhC could *technically* play a role of a watchful, responsible and responsive carer, it lacks the core qualities that are inalienably-human, such as emotion, empathy (described by Vallor (2011) as “an emotive/perceptual capacity to feel with another sentient being, to co-experience”), and devotion—inherent to and essential for caring *for* and *about* people. Also, is the care-recipient’s responsiveness to the care received from the AIhC productive within HRIs? For responsiveness to be truly constructive and trustworthy, evoking reciprocal reactions—it needs to be authentic in the human sense. But authenticity in HRI seems to be a misnomer [from a human perspective, at least]. As for reciprocity in the deeper sense of mutuality—although AIhCs, particularly through conversational “skills” can invoke a two-sided exchange between robot and care-recipient, this notion seems somewhat foreign to HRI. How is an AIhC to implement such non-translational/non-transferable values to fulfill these human-specific functions?

To conclude this point, arguably, the EoC components cited, are specific for human-human interaction, and therefore bound to be missing from HRI, or at least, require an adaptation to the latter.

And so, to compensate for these shortcomings of EoC with regard to robot-care, we need to consider a new derivative normative theory.

## From ethics of care to ethics of ‘Techno-care’

At the outset, we dub the (outsourced) care for humans by robots and smart technological devices—“techno-care”. Now, as we found no specific ethical frameworks, to date, relating to the use of AIhCs in healthcare, we suggest an “ethics of techno-care” (EtC) framework, to govern the design, development and use of AIhCs.

We recognise that to justify an EtC framework, we need to distinguish its care setting and care-relationships from those of EoC. First, unlike EoC applied to the caregiver-care-recipient binary relationship, EtC concerns a triangular relationship between the person in need of care, a supportive robotic system, and the human (professional/family-member) caregiver implementing the system (Agraz et al. 2022). This has direct bearings on responsibility allocation and accountability expectations, which potentially apply to a number of human actors (professional caregiver, AIhC programmer, AIhC deployer) and the artificial agent, the robot, if considered a moral agent. We shall briefly address it below.

Second, while within this new kind of [human-robot] relationship, some sort of care *is* being provided by AIhCs, techno-care retains elements of the act of caring per se, while lacking the human-centred features and qualities of caring, particularly as they relate to the care-provider’s (AIhC) authentic (human-like) responsiveness and motivation, as argued above. For instance, the EoC feature of “impulse to realise care” as dubbed by Maio (2018), can be represented in techno-care as a mere technical prompt to initiate an act of care. Similarly, core fundamentals of EoC, such as the reciprocity between care-provider and care-recipient, should be differently construed when being cared for by robots, with the expectation for meaningful reciprocity—somewhat attenuated.

Third, an EtC framework, being equally about dependencies between caregiver and care-recipient as EoC, would reflect looser forms of relationship/attachment between the robot care-provider and the human care-recipient. Maio (2018) presents (as a limitation of care ethics) that “[s]ometimes a patient has no desire to enter into a relationship, but simply wants to make use of a service”. Realistically, AIhCs provide *a service of caring*, and not caring per se. And so, the anthropology of dependence also takes on a different form, as care-recipients’ reliance on AIhC-support is rather instrumental (although they may develop emotional attachment to the robot).

Last, but perhaps most importantly, EoC provides ethical guidance for human caregivers within human-human interactions. The ability to develop and train carebots that are capable of ethical behaviour has encountered a sceptical attitude (Sharkey 2017), which we share. It seems unlikely that robots could be similarly guided by being internally coded with an EoC-like ethical, universal rules in the apparent future (Sharkey and Sharkey 2021). EtC is therefore warranted as guidance for programmers and implementers, for AIhC design, implementation and ethical regulation. Notably, EtC principles, as we conceive them, will likely draw on elements from all ethical frameworks discussed here, interwoven to form a new normative guidance. First and foremost, this framework will be inspired by EoC principles, *mutatis mutandis*. Also, relevant care-recipient-centric principles of bioethics, such as autonomy, human dignity and non-maleficence—will be applied to the personal and societal aspects of implementing AIhCs. AI ethics human-centric principles (e.g., transparency and explainability), will be applied to the general framework and technological architecture of AIhC; and roboethics principles will be applied to the setting of techno-care, to ethically guide human-care-recipient–robot-care-provider interactions.

Our proposed EtC framework should incorporate the following key elements:

- Human care-sensitive design. EtC will incorporate a commitment to human-sensitive care in its design (within the limits of robots), particularly in terms of (techno-)attentiveness, reciprocity, dignity (for example, an AIhC assisting a care-recipient with (un)dressing can be programmed, according to Western etiquette, to turn its head away to ensure that the latter feels comfortable, with her privacy maintained and dignity—intact). This should be commensurate with the degree of the care-recipient’s vulnerability (i.e., the more vulnerable the care-recipient—the more nuanced is the sensitivity required). Recognising that this is challenging for programmers (i.e., the *you-cannot-program-humanness-argument*), this boundary needs to be pushed farther as technology advances and as society will become more habituated to AIhC-care [*the new* (dystopic?) *normal*].
- Minimally-necessary deception. The instrumental use of deception in care, and its (case-dependent) conditional justification (i.e., to the extent required to accomplish the care-recipient’s well-being) are unique for techno-care settings, whereas deception is considered unethical under EoC principles. It may be impracticable, even undesirable in some cases, to avoid deception in AIhCs (Sharkey and Sharkey 2021). Its justification will require weighing ethical trade-offs. And so, if AIhC-deception is necessary to achieve better health and wellbeing outcomes for the care-recipient and does not pose greater harms to her dignity and autonomy than those envisaged by human caregivers—it could be acceptable.
- Techno-care transparency—to ensure care-recipients’ awareness to being cared for by a non-human caregiver. This should encompass explanation (to the care-recipient, or surrogate decision-maker) about the AIhC’s mission (whether it assists a human caregiver, or replaces it) and degree of artificial autonomy, the specific care functions it offers, the nature of HRI and its limitations (e.g., *techno-*attentiveness), etc.
- Techno-care privacy design, to limit AIhC-data collection to the minimum required to provide safe and effective care, with nuanced consent options (e.g., collecting health data, video and audio data, or any combination thereof). Also, the collection of the data must be in direct relation to the care-recipient’s wellbeing and cannot be used for any secondary purposes, such as research, neither shared with vendors or deployers, without the care-recipient’s or its surrogate decision-maker’s consent.
- Techno-care autonomy. Namely, respecting the care-recipient’s values, preferences and volitions as expressed by them, or as previously known (if cognitively impaired), in the context of care. It specifically requires the care-recipients’, or surrogate decision-makers’ informed consent to be cared for by an AIhC, within a specific HRI. EtC-informed consent will address the issues mentioned in (c) and (d) above, as well as AIhC’s potentialities, and alternatives to AIhC-care. Sharkey and Sharkey (2012) and Sorrel and Draper (2014) importantly observe that the carebot’s autonomy-respecting features, could occasionally be constrained by the need to protect care-recipients’ physical welfare. For instance, the robot not respecting a care-recipients suicidal request, which “there would be a good moral reason for programming a carebot not to comply with” (Sorrel and Draper 2014). Techno-care autonomy also uniquely calls upon creators and implementers of AIhCs, to circumspectly balance a carebot’s artificial autonomy with the autonomy of the care-recipient.
- Techno-care responsibility and accountability. Applying both to programmers and implementers, and to AIhCs, this involves determination of the moral agenthood of the robot itself, which is a contentious issue. For reasons of space, we shall only briefly address it here. Floridi (2014) defines moral agents as “any *interactive*, *autonomous* and *adaptable transition systems* that can perform *morally qualifiable actions*,” namely, cause moral good or evil. Seemingly, under robots’ moral agency-“enthusiasts”, such as Floridi and Sanders’ (2004; Floridi 2014), most AIhCs *technically* meet the criteria for moral agenthood (dependent on a certain level of abstraction), including: their ability to respond to environmental stimuli (e.g., such created by care-recipients); their autonomy in the sense of being able to change their states according to their ‘transition rules’, in a self-governed way, independently of said environmental stimuli (flexible decision-making in care); and their adaptability, reflected in their ability to change the transition rules themselves (modifying the provided care according to data about treatment success/failure). But we are not convinced that these “technical checks” are sufficient. Other scholars, crudely described as robots’ moral agency-“sceptics”, put emphasis e.g., on robots being non-living machines that “lack… a biological substrate”, necessary for the development of morality (Sharkey 2017); or acknowledge computer systems as moral entities but not as moral agents,^8^ as they do not have (human-like) mental states, nor intendings to act arising from an agent’s freedom (Johnson 2006).

But realistically, we are not there yet. The gap between AIhCs present abilities and recognising them as legal entities with full moral agency, entails that human actors—i.e., developers and deployers, must assume a complementary (human) responsibility, as well as be held accountable for any harms that AIhC-care may incur. This is consistent with Floridi’s (2014) position that in cases where an artificial agent is being qualified as a moral agent—it “does not relieve the creator of that agent of responsibility”. He stresses that what counts is their *moral accountability*, as responsibility should lie with their developers. Similarly, Sharkey (2017), advocating a responsible robotics approach, holds that “[h]umans should not offload their responsibility for the effects of robot actions onto the robots,” and that robots’ involvement in morally sensitive situations ought to be limited. However, this aspect of AIhCs’ answerability is a dynamic one, and may very well change if/when a robot is recognised as a moral and legal entity in itself. Until such time comes—accountability will remain with humans and other legal entities (e.g., AI companies) involved.

(g) Diversity and cultural sensitivity in AIhC design. These are significant for promoting inclusiveness among care-recipients, and for effective cross-cultural communication, i.e., for care-recipients from different ethnic backgrounds to confidently connect with AIhCs and comply with the care offered by them. A cultural-sensitive design relates to both *facial features* (semblance to various population groups) and to culture-specific *facial expression signals* of the humanoid (Jack 2013). AIhC’s cultural-sensitive design, could allow for culturally personalised care, and also promote global justice in healthcare, if deployed in developing countries, in the future.

Notably, however, such culture-sensitive task could be challenging for the following reasons: (a) the complexities of emotion communication; (b) having prominently Western developers; (c) relying on non-representative data, typically that of Western facial expression signals (neglecting those of other cultural groups); (c1) lack of experience in processing other-race faces, potentially leading to a lack of expertise in decoding (subtly variable) culture-specific facial expression signals; and (d) the universality of facial expressions being a contentious issue in itself (as some scholars argue for cultural *specificity*) (Jack 2013).

To conclude, we presented here a non-exhaustive, initial framework of EtC. While, naturally, there are many parallels between the frameworks of EoC and EtC, we attempted to illustrate some distinctive features of the latter, built on very different relationships between human and machine.

## Conclusion

Truth be told—it does not look like the hegemony of humans in human care is over. The human-unique qualities of care, or what we termed as the *you-cannot-program-humanness-argument*, remain a significant challenge for AIhC developers and deployers.

Conceivably, the real challenge is accepting that this is unachievable, or to forego trying altogether, cognisant that a new kind of care—*techno-care*, is emerging. And perhaps, given the shortage in human caregivers, this newly forged relationship between human and robot, should not be outright dismissed. Particularly, as testimonies of users of social robots, indicate that some sort of human-like relationship, *is* being formed.

And so, without getting into questions of social justice, one could envision future health insurances offering two alternative models of support care services—the more costly human care, and the more affordable—techno-care.

But before moving from lab to practice, the implementation of AIhCs in healthcare and welfare settings should be tested and validated in randomised controlled trials, a hurdle which most such applications have not yet crossed (Fiske et al. 2019). Observational research and qualitative interviews with care-recipients experiencing the implementation of AIhCs are also needed, to properly understand and socio-ethically evaluate the deeper meanings and effects of human-AIhC interactions.

We recognise that the present lack of empirical validation, and the theoretical nature of this paper, may somewhat limit its practical applicability at this stage. Nonetheless, we believe that our proposed EtC framework, provides a much-needed ethical compass to guide developers, implementers and policymakers, both presently and even more so, once the empirical gap is overcome to a certain extent.

## Footnotes

1 Albeit their framework is limited to carebots for older people *with* capacity and does not address humanoid carebots in the sense put forward in this paper—i.e., robots designed with human facial expressions and posture, simulating human behaviour.

2 This should be read as deception in a soft (not necessarily ill-intentioned) sense.

3 In response to this, some may argue that AIhC-deception is not meaningfully different from, say, a patient with dementia who mistakes a human nurse for their daughter. While such a patient could be said to be similarly deceived, deeming such “deception” as ethically impermissible—would seem implausible. But the two settings are distinct in the intentionality of their deception, yielding different moral judgments. In the case of AIhCs, deception is a deliberate act (by design), external to the care-recipient, whereas the nurse/daughter scenario is a case of an internal, auto(self)-deception, namely, an unintentional result of the patient’s condition, rather than a purposeful act to achieve a therapeutic/care goal. So, while the ethicality of AIhC-deception is contentious as we briefly analyse here, the latter case of (self-) deception, seems beyond ethical reproach (for the patient, at least. The ethicality of the nurse’s conduct, whether refuting her identity as the patient’s daughter, or keeping the delusion alive—is another matter, beyond the scope of this paper).

4 AIhC’s inherent deception could also be argued as paternalistic, particularly for vulnerable care-recipients who are dependents, and may prefer human care but cannot effectively express objection to be cared for by an AIhC. Conversely, according to Coin and Dubljević (2021) it could be equally paternalistic and stereotypical to wholly dismiss elderly judgement (including those not meaningfully cognitively impaired), and to simply assume that they can be easily tricked by the robot’s anthropomorphic features into thinking that they are being reciprocated with genuine emotion and empathy by the carebot.

5 Another example is the OECD (2019) AI Principles, incorporating values such as transparency and explainability; as well as respect for human-centred values and fairness, including autonomy of individuals and dignity.

6 A fourth law was later added by Asimov (1985; Encyclopaedia Britannica n.d.).

7 See e.g., (European Parliament 2019; European commission 2019).

8 Notably, Johnson’s perception, formulated nearly two decades ago, may not fully apply to advanced autonomous machine learning-based AI systems, such as AIhCs.
